# Emotional well-being and gut microbiome profiles by enterotype

**DOI:** 10.1038/s41598-020-77673-z

**Published:** 2020-11-26

**Authors:** Sung-Ha Lee, Seok-Hwan Yoon, Yeonjae Jung, Namil Kim, Uigi Min, Jongsik Chun, Incheol Choi

**Affiliations:** 1 Center for Happiness Studies, Seoul National University, Seoul, Republic of Korea; 2 ChunLab, Inc., Seoul, Republic of Korea; 3 School of Biological Sciences, Institute of Molecular Biology and Genetics, Seoul National University, Seoul, Republic of Korea; 4 Department of Psychology, Seoul National University, Seoul, Republic of Korea

**Keywords:** Microbiology, Psychology, Gastroenterology

## Abstract

With increasing attention being paid to improving emotional well-being, recent evidence points to gut microbiota as a key player in regulating mental and physical health via bidirectional communication between the brain and gut. Here, we examine the association between emotional well-being and gut microbiome profiles (i.e., gut microbiome composition, diversity, and the moderating role of the enterotypes) among healthy Korean adults (*n* = 83, mean age = 48.9, SD = 13.2). The research was performed using high-throughput 16S rRNA gene sequencing to obtain gut microbiome profiles, as well as a self-report survey that included the Positive Affect Negative Affect Schedule (PANAS). The cluster-based analysis identified two enterotypes dominated by the genera *Bacteroides* (*n* = 49) and *Prevotella* (*n* = 34). Generalized linear regression analysis reveals significant associations between positive emotion and gut microbiome diversity (*Shannon Index*) among participants in the *Prevotella* dominant group, whereas no such relationship emerged among participants in the *Bacteroides* group. Moreover, a novel genus from the family *Lachnospiraceae* is associated with emotional well-being scores, both positive and negative. Together, the current findings highlight the enterotype-specific links between the gut microbiota community and emotion in healthy adults and suggest the possible roles of the gut microbiome in promoting mental health.

## Introduction

Recent findings suggest that the neuroendocrine-immune pathway connects the brain and gut microbiota—known as the brain-gut-microbiota axis. Given that growing evidence points to the substantial relationship between the gut microbiota and a number of complex human behaviors, the gut microorganisms community is emerging as a key mechanism for modulating mental well-being. In fact, emotional disorders—such as depression and anxiety—are frequently accompanied by functional gastrointestinal disorders, suggesting an association between gut function and psychiatric diseases^1,2^. In addition to these observations, findings from a longitudinal panel further suggest that intestinal infections significantly predict the future onset of anxiety disorder^3^. Although the mechanisms underlying the association between gut microbiota and mental health are still under investigation, recent rodent studies reveal that alterations in gut microbiota significantly modulate emotional behaviors, such as depression, anxiety, and stress-related behaviors^4–6^. There is also some clinical evidence indicating the significant links between the gut and emotion; for example, altered gut microbiota composition was reported in patients with major depressive disorder (MDD) in terms of fecal microbial diversity (estimated by the *Shannon index*) as well as the level of the genus *Faecalibacterium*^7^. In healthy adults, favorable personality types (i.e., high in openness and conscientiousness) and self-rated higher quality of life were associated with the composition of certain gut microbiota (i.e., *Faecalibacterium*, *Coprococcus*, and *Lachnospiraceae*) as well as an enriched diversity of the gut microbiota community^8,9^.

Expanding volumes of studies on “psychobiotics” also support the significant link between gut microbes and mental well-being. Psychobiotics includes a family of beneficial bacteria, as well as dietary soluble fibers which stimulate the growth of “good” bacteria^10–12^ that enhance mental well-being. Again, given the high comorbidity present between mental health issues and irritable bowel disorders (IBS), it is plausible that the modification of microbial ecology via an intake of psychobiotics could improve an individual’s emotional well-being. Recent preclinical studies support this idea, pointing to the impact of beneficial microbiota on well-being; in rodent models, treatment with probiotic bacteria significantly improved emotional behaviors^4^ as well as increasing the level of serotonin precursor, suggesting that probiotics have a potential role as an antidepressant^13^. In humans, the intake of fermented food containing probiotics also significantly improved positive affects with changes in emotion-related brain activation^14^. Similarly, the intake of probiotics decreased social anxiety and symptoms of depression, especially among those with high levels of neuroticism^15^. Moreover, the probiotic food-supplement intervention reduced negative thoughts associated with sadness among healthy volunteers^16^. Thus, the existing findings indicate that the modification of the gut microbiome by probiotic administration, or via food supplements, may closely affect one’s mood.

With emerging evidence of the significant links between gut microbes and emotion, potential moderating factors for its physiological adjustment are attracting increasing attention. Recent studies suggest that enterotypes have a moderate role in regulating the association between the gut microbiome and mental health^9,17^. Enterotypes refer to robust stratified clusters based on the variation found in the levels of one of three genera in the gut: *Bacteroides* (enterotype 1), *Prevotella* (enterotype 2), and *Ruminococcus* (enterotype 3)^18^. Which enterotype is present depends on, in part, the hosts’ long-term diet – i.e., the amount of ingested animal protein/saturated fats (*Bacteroides* type) versus carbohydrates/simple sugars (*Prevotella* type) – whereas they are less influenced by the hosts’ body mass, age, and sex^18–20^. Recently, a brain imaging study found distinct patterns of the emotional process and brain connectivity between stratified enterotypes; clusters with a greater abundance of *Prevotella* show higher levels of emotional response, along with prominence in the connectivity of emotional, attentional, and sensory processing brain regions when compared to *Bacteroides*-dominant clusters^17^. A large-scale microbiome study also revealed that the *Bacteroides*-enriched enterotype is significantly associated with a lower score on the subjective feeling of quality of life, as well as a higher score regarding depressive symptoms^9^. Although these previous studies suggest the possible role of enterotypes on mental well-being, more evidence is needed in order to conclude the definitive role played by enterotypes.

With the expanding interest in the communication between the gut microbiome and mental health, this nascent field still needs to build up empirical evidence. Moreover, although increasing evidence suggests an association between gut microbiome profiles and mental health, evidence to support the link between them, especially regarding the role of enterotypes, is understudied. To this end, this study assesses gut microbiota profiles along with the emotional well-being indicators of healthy Korean adults. We aim to identify whether or not emotional well-being is associated with gastrointestinal microbiota profiles, such as composition and diversity. Moreover, based on recent findings implying differential patterns of mental health by enterotype, we investigate whether the association between gut microbiota profiles and mood state differs by enterotype. In this exploratory study, we hypothesize that the gut microbiome profiles are associated with emotional well-being states, focusing specifically on the differential role by enterotype.

## Results

### Stratification of the participants based on their gut microbiome

**Figure 1:**
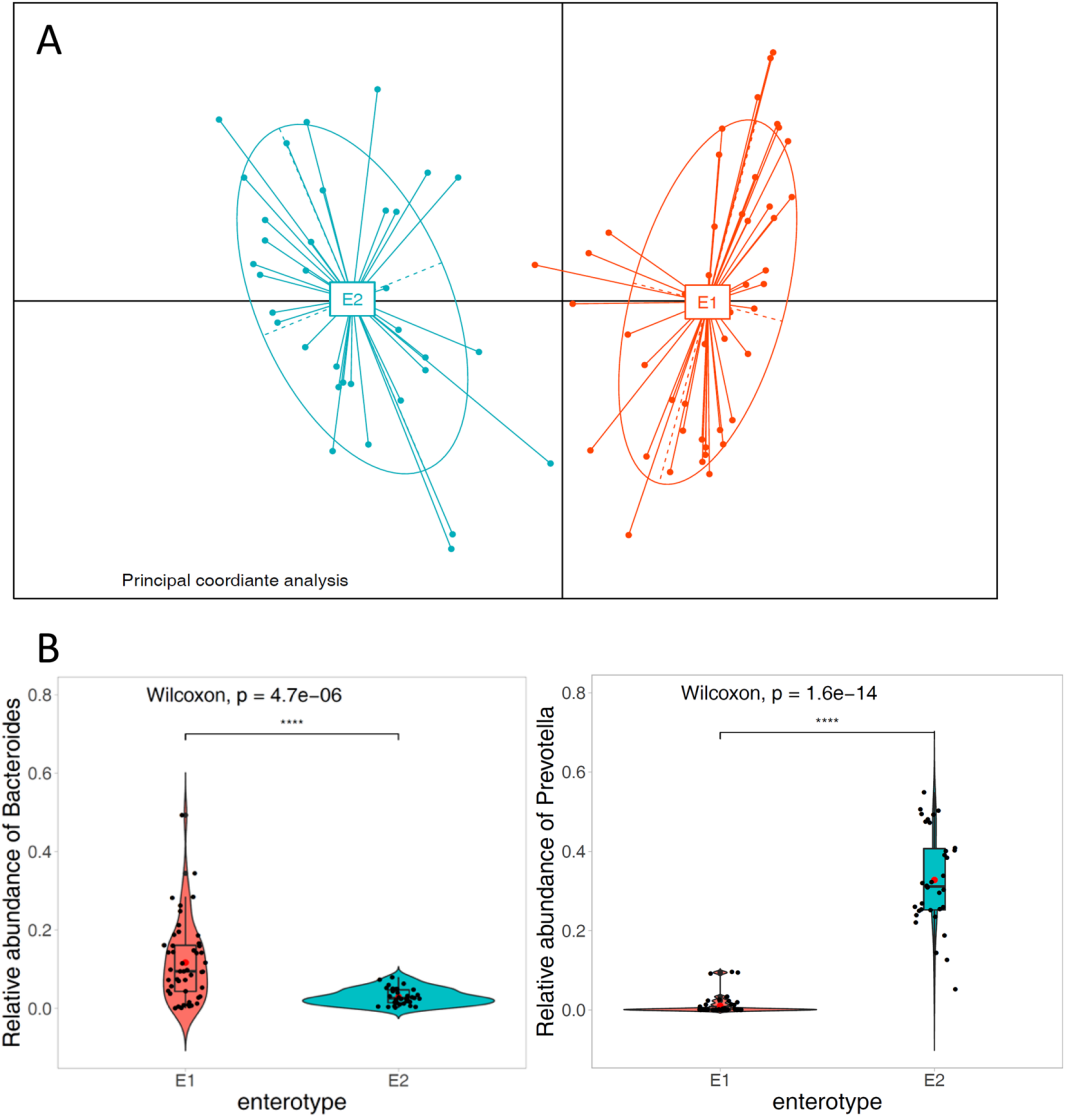
Identification of the two microbiota clusters. **(A)** The first two principal coordinates of the Jensen-Shannon distance of the microbiota (genus level) abundance profiles. Different colors represent enterotypes classified by the partitioning around medoids (PAM) clustering algorithm. **(B)** The relative abundance of *Bacteroides* and *Prevotella* in each enterotype.

Based on the microbiota composition, principal coordinate analysis (PCoA) revealed two distinct clusters, E1 and E2 (Fig. 1). In cluster 1 (E1) (marked in red in Fig. 1), *Bacteroides* were significantly more abundant than in cluster 2 (E2) (*p* < 0.001, Wilcoxon test), whereas in cluster 2 (marked in blue in Fig. 1), *Prevotella* was significantly more abundant in cluster 1 (*p* < 0.001, Wilcoxon test). The LEfSe analysis also confirmed the significant differential patterns for gut microbioal abundance in each cluster (Figure S1). Overall, 49 participants were included in the E1 (or *Bacteroides* group) and 34 participants in the E2 (or *Prevotella* group), respectively (Table 1). Statistical analysis of group differences revealed that significantly more female participants were present in the *Bacteroides* group, whereas male participants were more prevalent in the *Prevotella* Group (Table 1). No statistical differences between the microbiota clusters were found in terms of age, BSS scale, food preference (meat vs. vegetable), or degree of abdominal pain. Importantly, no differences were found between the groups regarding their emotional well-being scores—in terms of both positive and negative affect.

**Table 1:** Participants characteristics classified by cluster, Mean (SD) or Number (%).

	*Bacteroides*-high group (N = 49)	*Prevotella*-high group (N = 34)	Total (N = 83)	*p* value^1^
Age	48.1 (12.7)	50.0 (14.0)	48.9 (13.2)	0.515
**Gender**				0.009
Male	16 (32.7%)	21 (61.8%)	37 (44.6%)	
Female	33 (67.3%)	13 (38.2%)	46 (55.4%)	
**Antibiotics**				0.674
No	38 (77.6%)	25 (73.5%)	63 (75.9%)	
Yes	11 (22.4%)	9 (26.5%)	20 (24.1%)	
**BSS**^a^				0.462
1	2 (4.1%)	0 (0.0%)	2 (2.4%)	
2	6 (12.2%)	3 (8.8%)	9 (10.8%)	
3	7 (14.3%)	5 (14.7%)	12 (14.5%)	
4	27 (55.1%)	20 (58.8%)	47 (56.6%)	
5	7 (14.3%)	4 (11.8%)	11 (13.3%)	
6	0 (0.0%)	2 (5.9%)	2 (2.4%)	
Abdominal pain^b^	2.245 (1.974)	2.000 (2.335)	2.145 (2.119)	0.608
Food choice^c^	6.000 (2.131)	6.088 (2.610)	6.036 (2.324)	0.866
Positive affect^d^	3.329 (0.555)	3.203 (0.546)	3.277 (0.552)	0.311
Negative affect^d^	2.096 (0.738)	2.135 (0.783)	2.112 (0.752)	0.816
BMI^e^	24.364 (4.187)	24.739 (3.091)	24.524 (3.738)	0.686

^1^*p*-values were calculated from independent sample t-test or chi-square test (categorical variables; gender, BSS, antibiotics use).

^a^Bristol Stool Scale: 1 (hard lumps) to 7 (entirely liquid).

^b^0 (not at all) to 10 (severe) scale.

^c^Preference between meat versus vegetable: 1 (meat preferred) to 10 (vegetable preferred).

^d^1–5 scale.

^e^Based on a subset of the population (n = 68).

### Gut microbiome diversity by enterotype

**Figure 2:**
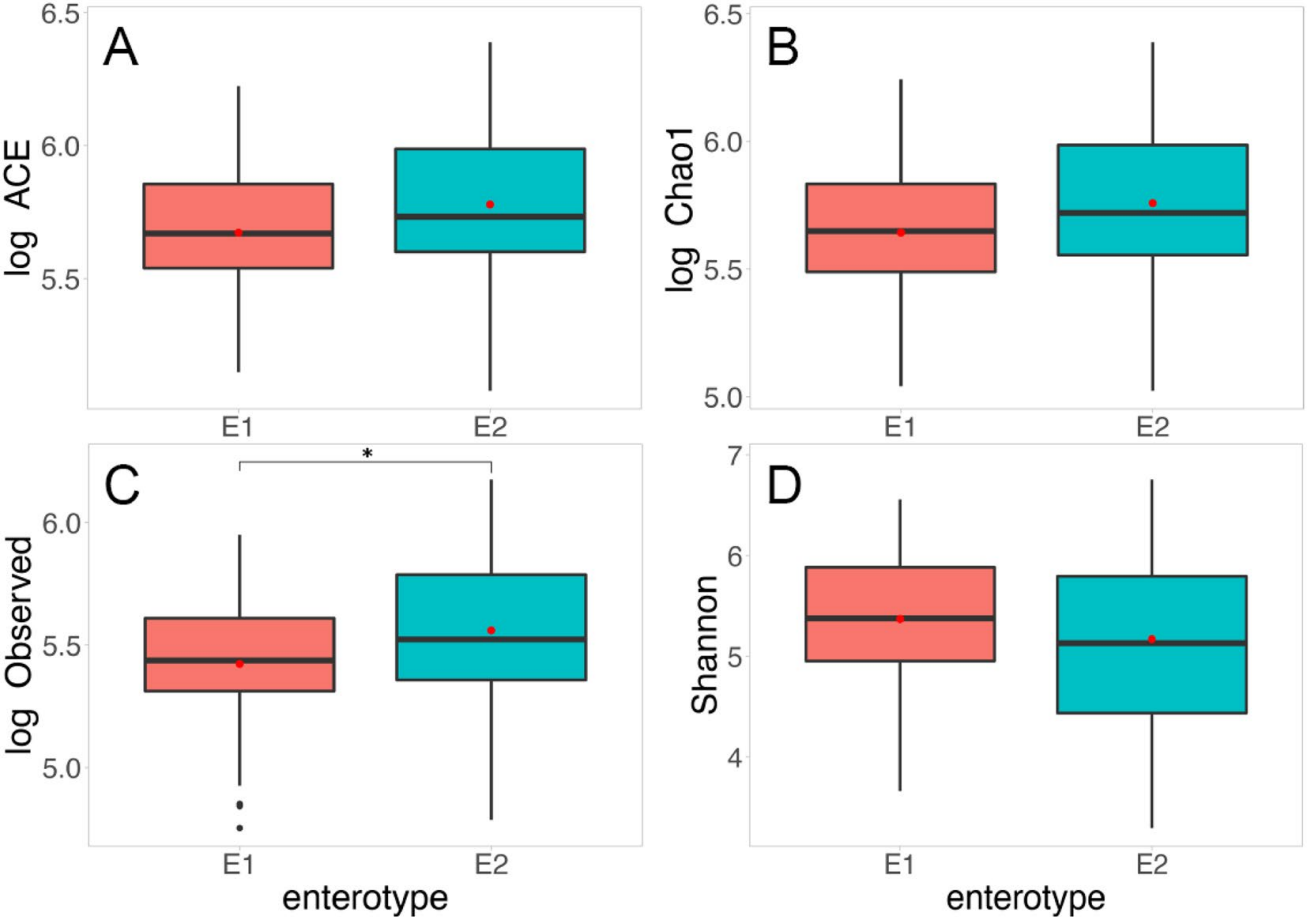
Comparisons of gut microbiome diversity indices; ACE **(A)**, observed **(B)**, Chao1 **(C)** and Shannon diversity index **(D)** classified by enterotype. *p < .05 (independent sample t-test). E1 = *Bacteroide*s group, E2 = *Prevotella* group.

To examine whether the diversity in gut microbiota species (Observed, Chao1, ACE, and Shannon Diversity Index) differ by enterotype, we performed an independent sample t-test, in addition to linear regression. To reduce the skewness in the distribution, Observed, Chao1, and ACE were log-transformed as they did not follow normal distributions. The *Prevotella* group, when compared to the *Bacteroides* group, demonstrates significantly higher in terms of the Observed index (t (81) = − 2.12, *p* = 0.037) as well as marginally higher in Chao1 (t (81) = − 1.94, *p* = 0.056) and ACE (t (81) = − 1.81, *p* = 0.074) (Fig. 2). Moreover, the generalized linear model further confirmed that the *Prevotella* group showed significantly higher species richness scores after controlling for age, sex, and antibiotics use (Chao1: *b* = 0.14 (reference group = E1), SE = 0.06, *p* = 0.026; ACE: *b* = 0.13 (reference group = E1), SE = 0.06, *p* = 0.031; Observed: *b* = 0.17 (reference group = E1), SE = 0.07, *p* = 0.011). However, the Shannon diversity index did not differ across the two enterotypes when using either the independent t-test (t (81) = 1.17, *p* = 0.24) or linear regression model (*b* = − 0.12 (reference group = E1), SE = 0.17, *p* = 0.479).

### Gut microbiome and positive/negative emotion

More importantly, we further examined the association between the gut microbiota diversity and emotional well-being scores measured by PANAS, as well as the moderative role of the enterotype. First, we examined the relationship between emotional well-being (either positive or negative emotion) with the four gut microbiome diversity indices. Among these indices, the Shannon diversity index was significantly associated with the positive affect scores, but not with the negative affect scores (positive affect: *b* = 0.306, SE = 0.15, *p* = 0.045; negative affect: *b* = − 0.114, SE = 0.112, *p* = 0.312). The remaining three diversity indices did not show any significant association with emotional well-being scores. When we considered the interaction term between the positive affect scores and enterotypes via the linear model, the interaction term and the enterotype became statistically significant (positive affect × enterotypes: *b* = 0.618, SE = 0.30, *p* = 0.042; enterotype: *b* = − 2.08, SE = 0.98, *p* = 0.037, respectively).

**Figure 3:**
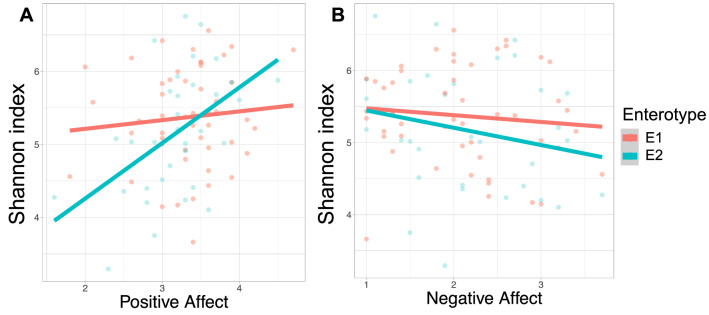
Relationship between the Alpha diversity (observed Shannon diversity index) and positive affect **(A)** and negative affect **(B)** in the two microbiota clusters (red line—E1, *Bacteroides* cluster; blue line—E2, *Prevotella* cluster).

To further examine the association between the Shannon diversity index and the PANAS scores in relation to enterotypes, we further investigated the association between the positive / negative affect scores and the Shannon index for each enterotype. As a result, in the *Prevotella*-predominant group (E2), the positive affect scores were significantly associated with the increased level of Shannon diversity index (*b* = 0.61, SE = 0.28, *p* = 0.039, Fig. 3A; Figure S2 A for the predicted outcomes), whereas the negative affect scores did not show any association (*b* = − 0.10, SE = 0.19, *p* = 0.603, Fig. 3B; Figure S2 B). On the other hand, in the *Bacteroides*-predominant group, (E1) neither the positive nor negative affect scores were associated with the Shannon diversity measures (positive affect: *b* = 0.049, SE = 0.172*, p* = 0.775; negative affect: *b* = − 0.167, SE = 0.132, *p* = 0.212).

Moreover, in order to examine the possible long-term effect of positive affect on the Shannon index, we examined the association between the positive affect scores collected one and a half years before the stool samples from the same participants. The positive affect scores obtained from Wave 1—measured approximately one and a half years ahead of the stool collection—were associated with the Shannon index; again specifically, the *Prevotella* group showed a similar pattern of association between the positive affect scores and the Shannon index (*b* = 0.48, SE = 0.27, *p* = 0.087), whereas the *Bacteroides* group did not show any association (*b* = − 0.008, SE = 0.14, *p* = 0.95). The similar patterns between the two positive affect scores—at time of the stool collection and one and a half years ahead of the time—seem to be largely due to the stability of positive affect (according to the Pearson correlation between the current positive affect and Wave 1 positive affect scores, where *r* = 0.41; *p* < 0.001).

**Figure 4:**
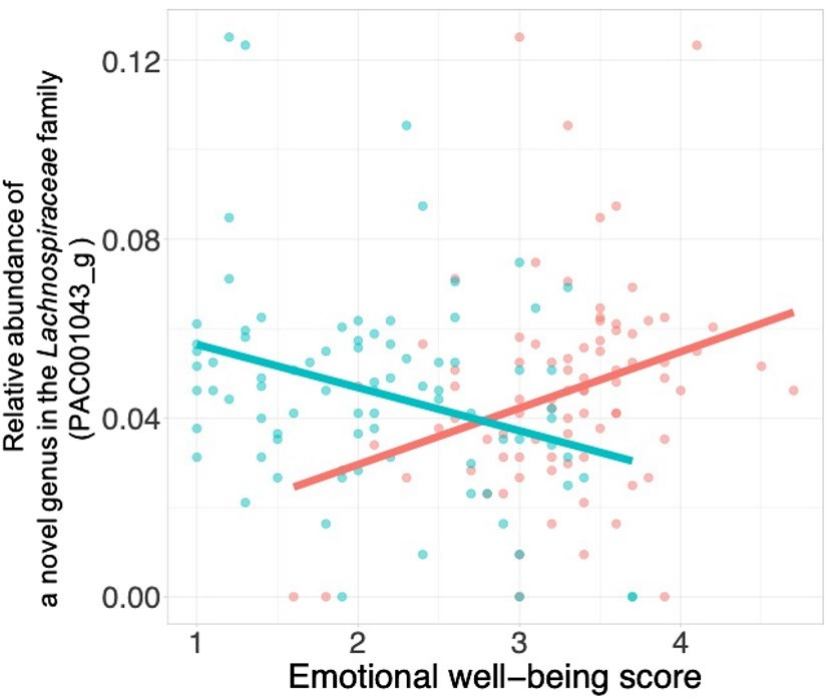
The association between the emotional well-being scores and abundance of gut microbiome (a novel genus in the *Lachnospiraceae* family PAC001043_g taxa) in the total population. The abundance of PAC001043_g taxa was associated with a decreased level of the negative affect (blue line), and was related to an increased level of positive affect (red line).

Furthermore, to examine which specific microbial taxa are significantly correlated with mood status, we performed MaAsLin analysis with genus-level taxa present in at least 80% of the participants (the number of the taxa for the analysis = 52). A multivariate generalized linear regression analysis identified distinct sets of gut microbiota related to emotional well-being in the total population. Higher levels of *Agathobaculum* and *Collinsella* were inversely associated with the negative affect scores and the positive affect scores, respectively (*b* = − 0.014, SE = 0.005, *p* = 0.008, *q* = 0.23; *b* = − 0.051, SE = 0.015, *p* = 0.001, *q* = 0.095). Moreover, the abundance of PAC001043_g—a novel genus in the *Lachnospiraceae* family—was significantly associated with the higher positive affect scores and lower negative affect scores (positive affect: *b* = 0.014, SE = 0.005, *p* = 0.008, *q* = 0.214; negative affect: *b* = − 0.01, SE = 0.004*, p* = 0.01, *q* = 0.23) (Fig. 4; Figure S3).

## Discussion

The current study reveals that gut microbiome diversity is related to emotional well-being, and that enterotypes significantly moderate the links between emotional well-being and gut microbiome health (e.g., diversity). The enterotypes, which stratified the participants by gut microbiome profiles, did not alter mood status itself, but moderated the strength of the association between one’s mood and gut microbiome diversity. In the *Prevotella*-dominant group, emotional status was more closely related to gut microbiota diversity, such that positive affect was associated with increased gut microbiota diversity. However, in the *Bacteroides*-dominant group, one’s mood status was not significantly associated with gut microbiome diversity. This finding is in line with the results of Tillisch et al.^17^ in that only the *Prevotella*-high group displayed an increased response to affective images in the limbic system.

The current findings suggest a significantly tighter connection between “feeling good” (emotional well-being) and the well-being of the gut microbiota community, particularly in the *Prevotella-*dominant condition. Alterations in gut microbiomes were found in many mental diseases, including autism spectrum disorder, anxiety, depression, and schizophrenia^21, 22^. Among the various indicators of the gut microbiome, the microbiota diversity index is suggested to be a major marker for gut health as it reflects both ecosystem stability and resilience^23^. A few studies have linked candidate factors related to emotional well-being and gut microbiota diversity regardless of the enterotypes. For example, stress and anxiety significantly reduce gut microbiome diversity^24^. In addition, favorable social environments—such as higher socioeconomic status and social integration—have been linked to increased gut microbiota diversity^25,26^. Furthermore, psychological traits—such as openness (a personality categorized as the enjoyment of trying new things)—are also associated with increased gut microbiome diversity^8^. Collectively, both social and psychological factors that contribute to one’s mental well-being are known to be related to gut microbiome diversity. Since social and psychological factors influence emotional well-being, it is difficult to rule out the potential mediating effect of these factors on the association found within this study. Well-designed future studies can elucidate the direct and indirect links between emotional well-being and the gut microbiome society.

The current findings support the enterotype-specific link between emotional well-being and the gut microbiome community. The enterotypes help consider the complexity of the gut microbiome in a simpler way^27^. Even though there are discussions in which enterotypes were not distinct clusters—but showed a smooth gradient distribution of gut microbiota (i.e., not as clearly delimited as human blood groups)—classifying the characteristics of human microbiome variations into two or three groups (enterotypes) facilitates the understanding and application of the gut microbiome in medical fields (e.g., developing biomarkers based on enterotypes). Since no firm association has been established between enterotypes and any specific health outcomes or physiologic phenotypes^28^, the current findings contribute to the exploration of potential links between enterotypes and emotional processes. Furthermore, considering the stability of enterotypes over time, elucidating their connections with psychological traits (believed to be stable over time as well^29^) can be useful in developing candidate psychobiological markers that may have a long-term effect.

The current study finds that a few significant genera are associated with emotional well-being even though the exact functions have not been fully explored. For example, a novel genus from the *Lachnospiraceae* family is associated with both positive and negative emotion. Previous studies that individuals with major depressive disorder have significantly fewer *Lachnospiraceae*; *Lachnospiraceae* produces short-chain fatty acids (i.e. butyrate) which plays an important role in the brain-gut axis^7,30^. Moreover, regarding that the genus *Collinsella* has been associated with negative affect, one previous study reveals its connection with insulin levels in pregnant women^31^ and more studies are needed to examine its role in mood. Furthermore, the current study does not detect any significant link between well-known probiotic microbiota (e.g., *Bifidobacterium* and *Lactobacillus*) and emotional affects. Future research could further investigate the psychobiotic effects of novel genera found in current studies, as well as the effects of probiotics on one’s emotional well-being.

The present study has some limitations. First, although it reveals a significant relationship between gut microbiome profiles and positive/negative emotion in Korean adults, the cross-sectional design prevents us from drawing a causal relationship between emotional well-being and gut microbiome characteristics. Second, the limited number of participants in each enterotype group could have decreased the power of the association between gut microbiome profiles and well-being scores. Third, even though the enterotypes in the current study were classified using the PAM clustering method, enterotypes can be defined differently depending on the classification procedures (i.e. Dirichlet Multinomial Mixture modeling^32^). Lastly, due to the lack of information about actual health status (e.g., diabetes and obesity) and nutritional intake, we cannot rule out the influence of other confounding factors on the connection between emotional status and the gut microbiome.

In conclusion, the present study links one’s emotional status to gut microbiome diversity and composition. The results suggest that emotional behavior can be associated with gut microbiota profiles in healthy adults, especially when stratified by enterotype. The enterotype-specific links between emotional well-being and gut microbiome diversity suggest that enterotypes may work as an individually tailored intervention. Further investigations will fill the gap in our understanding of how gut microbiota communicate with the brain to affect emotions—in this case, the feeling of happiness. Moreover, future studies on enterotype-specific mood modulation will evaluate the suitability of the microbiome in offering a new therapeutic paradigm for mood disorders.

## Methods

### Participants

The participants were recruited from the Korean Adult Longitudinal Study (KALS), which recruited 561 Seoul residents between from 2017 and 2018 using random-digit-dialing of cell phone numbers with the age, gender, and geographical areas in Seoul equally distributed (Wave 1). A subset of the population (*n* = 85), who agreed to submit their stool samples, signed the consent form after fully explaining the purpose and procedures of the study. Subsequently, these participants completed the survey which included the Positive and Negative Affect Schedule (PANAS) and questionnaires concerning health conditions.

### Emotional well-being measures

Emotional well-being was measured using the Positive Affect and Negative Affect Schedule (PANAS)^33^. The PANAS contains 20 self-reported emotions: ten positive (e.g., interested, excited; Cronbach’s alpha = 0.86) and ten negative affects (e.g., afraid, nervous; Cronbach’s alpha = 0.92). Participants rated the extent to which they felt each emotion over the preceding week on a 5-point Likert scale (where 1 = “very slightly/not at all”; 2 = “a little”; 3 = “moderately”; 4 = “quite a bit”; 5 = “extremely”). The PANAS scores were obtained simultaneously with the stool collection and compared with the scores about one and a half years ahead of the stool collection.

### Behavioral and clinical measures

The participants self-reported their level of abdominal pain (on a scale from 0 = not at all to 10 = severe), their stool forms (Bristol Stool Scale; BSS)^34^, a 7-step categorical scale: 1 = separate hard lumps to 7 = entirely liquid), and the use of antibiotics in the preceding month. The dietary preference for meat versus vegetables by rating 1 (meat preferred) to 10 (vegetables preferred) was also collected.

### Stool collection

Participants collected the stool samples (~ 0.2 g) in SB-01 stool collection kits (ChunLab Inc., South Korea) that contained a lysis buffer (SDS 4%, Tris–HCL 50 mM, EDTA 50 mM, NaCl 500 mM). The samples were stored at room temperature until they arrived at the laboratory, which took less than three days. On arrival, the stool samples were stored in the laboratory at -80 ℃ until required to perform the PCR and further experiment.

### Gut microbiota analysis: isolation and 16S rRNA gene sequencing

After thawing fecal swabs and applying the homogenization process, the fecal suspensions were bead beaten and centrifuged at 14,000 *g* for 10 min. Supernatants were fused with nuclease-free water, and PCR amplification was performed. DNA amplification targets the V3V4 regions of the bacterial 16S rRNA gene using 341F and 805R primers. PCR products were sequenced using an Illumina MiSeq sequencing system (Illumina, USA) at ChunLab.

Classification and identification of 16S rRNA gene sequences for phylogenetic analysis were processed in EzBioCloud using the MTP (Microbiome Taxonomic Profiling) pipeline provided by ChunLab, Inc.^35^; the sequence quality was checked and low-quality reads (quality score less than Q25) were filtered out using Trimmomatic ver 0.32. After quality control, paired-end reads were merged using VSEARCH ver 2.13.4, followed by trimming the barcode, primer, and linker sites. Nonspecific sequences that did not encode 16S rRNA were detected using a hmmer in HMMER ver 3.2.1. Redundant reads were clustered after extracting unique reads using VSEARCH. Taxonomic assignment was performed by VSEARCH using a highly curated EzBioCloud DB^35^, which covers both valid and invalid names (phylotype). Chimeric sequences were detected using a reference-based approach via the UCHIME algorithm, using the non-chimeric 16S rRNA database from EzBioCloud. After taxonomic assigning and chimeric read filtering, reads below 97% similarity with the EzBioCloud database were de novo clustered using VSEARCH.

### Statistical analysis

Among the 85 samples, two samples were excluded because they did not meet the minimum count criteria (*n* = 10,000). All 83 remaining samples achieved sufficient coverage, as determined by Good’s coverage of > 99%. Copy numbers of the 16S rRNA gene were adjusted, and count normalization was performed with 11,289 (minimum count). The alpha diversity was analyzed with quantitative insights into microbial ecology (QIIME) after importing the species count matrix table, as obtained from the MTP pipeline^35,36^. The Shannon index measured species evenness, and Observed, Chao1, and ACE species richness estimators were measured. Since Observed, Chao1, and ACE do not follow normal distributions, they were log-transformed to reduce the skewness in the distribution.

Enterotype analysis was performed using PAM clustering (further information is available at http://enterotype.embl.de/enterotypes.html;Arumugam et al.^18^); the Jensen-Shannon Distance was obtained from the genus-level relative abundance matrix, and partitioning around medoid clustering was performed using the R ‘cluster’ package. The number of optimal clusters was set to two, with the maximum value of the Calinski-Harabasz (CH) index (CH index value = 60.450). Linear discriminant analysis of genus-level relative abundances, with full taxonomic hierarchy, was performed using linear discriminant analysis effect size (LEfSe), which compares differentially abundant taxa in groups using non-parametric tests. In each cluster, the association between emotional well-being scores and alpha diversity indices (Shannon index, Observed, Chao1, and ACE species richness) was calculated after controlling for possible confounding factors—including age, sex, and antibiotic treatment. Statistical analysis was performed using R (R version 3.6.3).

To examine the association between gut microbiome composition and emotional well-being, multivariate analysis was performed with the MaAsLin2 R package. Age, sex, and antibiotic use (taking antibiotics during the previous month, if “Yes” coded as “1”, if “No” coded as “0”), were controlled for as potential confounding variables. The resulting p-values were corrected for multiple comparisons using the Benjamini–Hochberg correction (FDR). A *p* < 0.05 and *q* < 0.25 (default values of MaAsLin package) were considered statistically significant.

### Ethical approval and informed consent

All procedures followed were in accordance with the ethical standards of the 1964 Helsinki declaration and its later amendments. The current study was approved by the Institutional Review Boards of Seoul National University (IRB No. 10-2018-21). Informed consent was obtained from all individual participants included in the study.

## Supplementary information

Supplementary Figures.
