# Supplementary material for: Emotional well-being and gut microbiome profiles by enterotype
Source: Sci Rep. 2020 Nov 26;10:20736. doi: 10.1038/s41598-020-77673-z (PMC7691370; doi:10.1038/s41598-020-77673-z)
Supplement: Supplementary file 1 — Supplementary Figures. [file 41598_2020_77673_MOESM1_ESM.docx]

**Supplementary information**

**Emotional well-being and gut microbiome profiles by enterotype**

**Sung-Ha Lee^1^, Seok-Hwan Yoon^2^, Yeonjae Jung^2^, Namil Kim^2^, Uigi Min^2^, Jongsik Chun ^2,3*^, Incheol Choi ^1,4*^**

^1^ Center for Happiness Studies, Seoul National University, Seoul, Republic of Korea

^2^ ChunLab, Inc., Seoul, Republic of Korea

^3^ School of Biological Sciences & Institute of Molecular Biology and Genetics, Seoul National University, Seoul, Republic of Korea.

^4^ Department of Psychology, Seoul National University, Seoul, Republic of Korea

**^*^** Correspondence concerning this article should be addressed to *Incheol Choi* (ichoi@snu.ac.kr) or *Jongsik Chun* (jchun@snu.ac.kr)


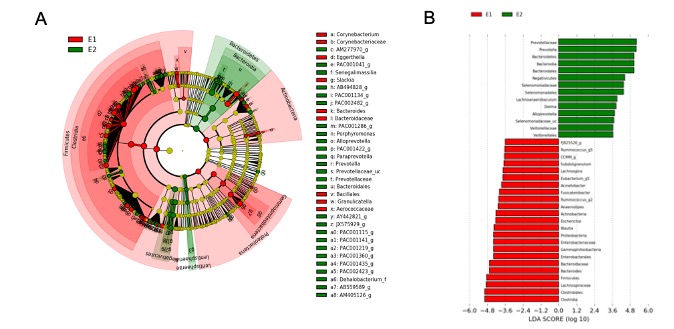


Figure S1. LDA effect size (LEfSe) analysis of the differences in gut microbial abundance between the enterotypes. A. Taxa highlighted on the phylogenetic tree (cladogram) using LEfSe with red and green colors indicate greater abundance in E1 and E2, respectively (A) The bar graphs also indicate the results from the linear discriminant analysis (LDA) score, differentially abundant taxa between the E1 and E2. Negative (red bars) LDA scores indicate the taxa more abundant in E1 whereas Positive (green bars) LDA scores indicate greater abundance in E2, respectively. Features with LDS scores >3.6 presented (B). The figures were generated using the Galaxy web application (https://huttenhower.sph.harvard.edu/galaxy).


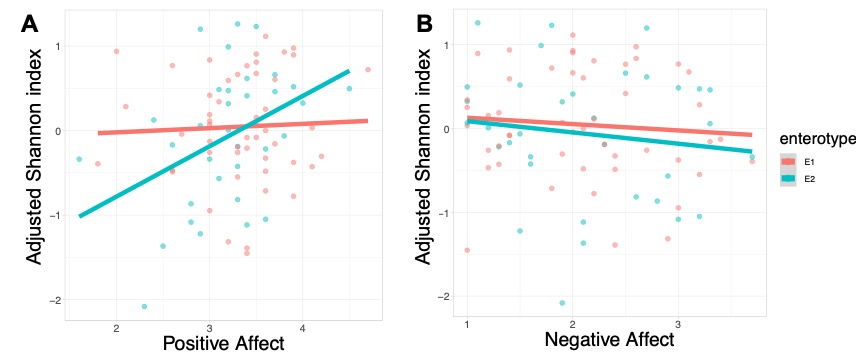


Figure S2. Adjusted Shannon index (the residuals obtained from the covariate model: Shannon index ~ age + gender + antibiotics use) and positive affect (A) and negative affect (B)


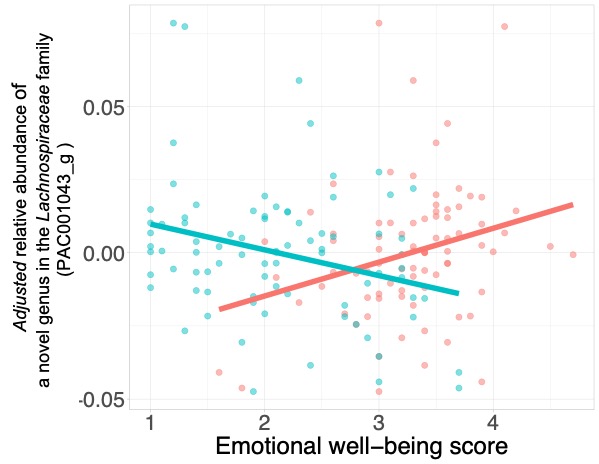


Figure S3. The association between the emotional well-being scores and an adjusted level of gut microbiome (a novel genus in the Lachnospiraceae family PAC001043_g taxa) abundance in the total population. The “adjusted” abundance of PAC001043_g taxa (the residuals obtained from the covariates model: relative abundance of PAC001043_g ~ age + sex + antibiotics use) was associated with a decreased level of the negative affect (NA; blue line), and was related to an increased level of the positive affect (PA; red line).
